# Oxidative Stress Markers and C-Reactive Protein Are Related to Severity of Heart Failure in Patients with Dilated Cardiomyopathy

**DOI:** 10.1155/2014/147040

**Published:** 2014-10-23

**Authors:** Celina Wojciechowska, Ewa Romuk, Andrzej Tomasik, Bronisława Skrzep-Poloczek, Ewa Nowalany-Kozielska, Ewa Birkner, Wojciech Jacheć

**Affiliations:** ^1^Second Department of Cardiology, School of Medicine with the Division of Dentistry, Medical University of Silesia, M. C. Skłodowskiej 10 Street, 41-800 Zabrze, Poland; ^2^Department of Biochemistry, School of Medicine with the Division of Dentistry, Medical University of Silesia, Jordana 19 Street, 41-808 Zabrze, Poland

## Abstract

*Background.* The aim of study was to determine relationships between functional capacity (NYHA class), left ventricle ejection fraction (LVEF), hemodynamic parameters, and biomarkers of redox state and inflammation in patients with dilated cardiomyopathy (DCM). *Methods*. DCM patients (*n* = 109, aged 45.97 ± 10.82 years), NYHA class IIV, and LVEF 2.94 ± 7.1% were studied. Controls comprised age-matched healthy volunteers (*n* = 28). Echocardiography and right heart catheterization were performed. Serum activities of superoxide dismutase isoenzymes (MnSOD and CuZnSOD), concentrations of uric acid (UA), malondialdehyde (MDA), and C-reactive protein (hs-CRP) were measured. *Results*. MnSOD, UA, hs-CRP, and MDA were significantly higher in DCM patients compared to controls. Except MDA concentration, above parameters were higher in patients in III-IV NYHA class or with lower LVEF. hsCRP correlated with of MnSOD (*P* < 0.05) and CuZnSOD activity (*P* < 0.01). Both isoenzymes positively correlated with mPAP and pulmonary capillary wedge pressure (MnSOD, resp., *P* < 0.01 and *P* < 0.05 and CuZnSOD *P* < 0.05; *P* < 0.05). UA positively correlated with MnSOD (*P* < 0.05), mPAP (*P* < 0.05), and PVRI (*P* < 0.05). The negative correlation between LVEF and UA (*P* < 0.01) was detected. *Conclusion*. There are relationships among the severity of symptoms of heart failure, echocardiographic hemodynamic parameters, oxidative stress, and inflammatory activation. Increased MnSOD activity indicates the mitochondrial source of ROS in patients with advanced heart failure.

## 1. Introduction 

Dilated cardiomyopathy characterized by alteration in the structure and function of the myocardium with dilation and impaired contraction of the left ventricle (LV) or both ventricles (LV ejection fraction <40%) and is sometimes called nonischemic cardiomiopathy.

Deterioration of myocardial performance resulting in the development of exercise intolerance and sometimes limited daily activity as symptoms of heart failure (HF) mostly assessed according to New York Heart Association (NYHA) Functional Classification [1]. There are a few points which were considered as a cause of dyspnea and fatigue:

1. hemodynamic parameters such as increased pulmonary capillary wedge pressure (PWP), pulmonary artery pressure (PAP), and pulmonary vascular resistance (PVR) [2];
2. the inspiratory and expiratory muscle weakness [3];
3. greater than normal phosphocreatine depletion and/or acidosis in the peripheral working muscle associated with impaired oxygen delivery [4];
4. mitochondrial dysfunction with impaired oxidative phosphorylation and defective electron transport chain at heart and skeletal muscles [4, 5].

One of hemodynamic features of dilated cardiomyopathy is increase of the pulmonary capillary wedge pressure (PWP) as indicator of elevated left ventricular filling pressure (preload). Up to 60% of patients with severe left ventricular (LV) systolic dysfunction may also present elevated pulmonary artery pressure due to passive transmission of elevated end diastolic pressure [6]. N-Terminal pro-B-type natriuretic peptide (NT pro-BNP) has become valuable biomarkers for confirming the diagnosis of HF correlated with hemodynamic abnormalities [7].

Left ventricle overload leads to a shift of fatty acid oxidation towards more efficient glucose oxidation. However, it also leads to reduction of maximal oxidative phosphorylation (OXPHOS) capacity with decreased activities of respiratory chain complexes and increase of electron leak [8].

Besides mitochondrial leakage reactive oxygen species (ROS) may arise from many sources, including vascular nicotinamide adenine dinucleotide oxidases [9], xanthine oxidases, autooxidation of catecholamines [10], and nitric oxide synthase activated by cytokines [11, 12]. Under physiological conditions their toxic effect can be prevented by such scavenging enzymes as superoxide dismutase (SOD), glutathione peroxidase (GSHPx), and catalase (CAT) as well as by other nonenzymatic antioxidants, but the elimination of ROS may be impaired because of decrease of antioxidant defense in human failing heart [13]. When the production of ROS exceeds the capacity of antioxidant defense, oxidative stress has a harmful effect on the integrity of biological tissue through lipid peroxidation cascades or direct oxidation of membrane proteins. Malondialdehyde (MDA) is one of the small-molecular-weight pieces resulting from the fragmentation of polyunsaturated fatty acids undergoing attack by ROS and is generally accepted index of lipid peroxidation [14].

Elevated circulating levels of inflammatory cytokines have been reported in heart failure, but most initial studies have focused on patient with cachexia or end stage failure (III and IV NYHA class) [15]. The further research have shown that serum concentrations of proinflammatory cytokines increase in patients as their functional heart failure classification deteriorates. Moreover, cytokines activation is unlikely to explain completely the association with neurohormonal activation [16]. Clinical and research studies revealed that low grade inflammation and also endothelial dysfunction in heart failure despite of its etiology may influence ROS formation [17, 18]. Recently a severe reduction in cardiopulmonary reserve and oxygen uptake efficiency concomitantly with an elevation of inflammatory biomarkers and prooxidative state in heart failure patients with preserved ejection fraction were found [19]. Moreover increased oxygen radical formation directly inside the human heart allografts was indicated but changes in the enzymatic antioxidative defense as adaptation to oxidative stress was biphasic and time limited [20].

We decided to assess potentially relationships between functional capacity, blood oxygenation, some echocardiographic parameters, invasive hemodynamic parameters, and serum biomarkers of reduction-oxidation reactions and inflammation in patient with heart failure due to dilated cardiomyopathy.

## 2. Study Group and Methods

### 2.1. Patients

We recruited 109 consecutive patients aged 18–80 years old with nonischemic dilated cardiomyopathy diagnosed according to the WHO criteria [21] who underwent right heart catheterisation (RHC) as routine assessment according to our heart transplantation protocol during hospitalization in our center between 01 January 2006 and 31 December 2011.

All patients were clinically stable; most of them received optimal conventional heart failure therapy including ACE inhibitor, *β*-blocker, mineralocorticoid receptor antagonist (MRA), digitalis, and diuretics for at least 1 month (Table 1).

Exclusion criteria included history of inflammatory musculoskeletal disorders, recent infection, any hemodynamic significant coronary artery stenosis assessed by coronary angiography, valvular heart disease, connective tissue disease, endocrine disorders, renal insufficiency, infectious disease, malignancy, and alcohol abuse.

The control group for hs-CRP, uric acid, MDA concentrations, and activity of superoxide dismutase isoenzymes measurements consisted of 28 healthy volunteers.

The study protocol was approved by the Bioethics Committee of Medical University of Silesia. Written informed consent was obtained from all enrolled patients.

### 2.2. Clinical Assessments

Noninvasive clinical assessment included physical examination, ECG, and echocardiography. The NYHA classification was used to assess functional capacity [1].

Echocardiographic images were acquired in standard views as recommended by the American Society of Echocardiography Committee. Left ventricular end-diastolic volume (EDV) and end-systolic volume (ESV) were obtained from the apical 4- and 2-chamber views by the modified Simpson's method. Left ventricular ejection fraction (LVEF) was calculated in a standard manner as follows: (EDV − ESV) × 100/EDV, to assess ventricular systolic function.

Right heart catheterization (RHC) was performed by the use of Swan-Ganz catheter (Star Edwards Lifesciences) administered under local anesthesia (1% Lignocaine) via the right jugular vein into pulmonary artery. Then two samples of mixed venous blood (SvO_2_) were collected in order to determine its saturation. After twenty minutes of stabilization of circulation parameters pulmonary wedge pressure (PWP), systolic pulmonary artery pressure (sPAP), diastolic pulmonary artery pressure (dPAP), and right atrium pressure (RAP) were measured. Cardiac output was measured by thermodilution using rapid bolus injection of 10 cc of cold saline. Systolic (sABP) and diastolic (dABP) systemic arterial pressure were measured noninvasively. Hemodynamic parameters were acquired five times—mean values were used for final evaluation. Acquired data enabled calculation of mean pulmonary artery pressure (mPAP) and mean systemic arterial pressure (mABP), pulmonary vascular resistance index (PVRI), and systemic vascular resistance index (SVRI).

- mPAP [mm Hg] equals the sum of dPAP and one-third of a subtraction of sPAP and dPAP in pulmonary artery (mPAP = dPAP + [sPAP − dPAP]/3).
- mABP [mm Hg] equals the sum of diastolic arterial blood pressure (dABP) and one-third of a subtraction of systolic arterial blood pressure (sABP) and dABP (mABP = dABP + [sABP − dABP]/3).
- PVRI [dyna*·*s*·*cm^−5^/m^2^] equals quotient of subtraction mPAP, PWP, and CI (PVRI = [mPAP − PWP]/CI 79.9).
- SVRI [dyna*·*s*·*cm^−5^/m^2^] equals quotient of subtraction mABP, RAP, and CI (SVRI = [mABP − RAP]/CI 79.9).

Blood pressure parameters were expressed in millimeters of mercury [mm Hg], CI as liters per minute [L/min/m^2^]. Resistance was expressed in dyna*·*s*·*cm^−5^/m^2^.

### 2.3. Biochemical Methods

Blood samples for laboratory assessments were obtained from the patients at time of RHC. Serum was separated by centrifugation at 1500 g for 10 minutes and was frozen at −70°C. Uric acid concentration was measured by colorimetric method (Roche, Cobas 6000e501). hs-CRP was determined in serum by ELISA method using commercially available kit. NT-proBNP was measured with the use of chemiluminescence method (Roche, Cobas 6000e501). Additionally, we also determined blood hemoglobin and serum creatinine concentrations using routine techniques.

SOD isoenzymes activity was determined with the use of spectrophotometric method by Oyanagui with KCN as the inhibitor of the CuZnSOD isoenzyme. CuZnSOD activity was taken as difference between total SOD activity and MnSOD activity. SOD activity was calculated against blank probe (containing bidistilled water). Enzyme activity was expressed as nitrite units (NU) per mL serum. One NU exhibits 50% inhibition of formation of nitrite ion under the method's condition [22].

Malondialdehyde was measured according to method described by Ohkawa using the reaction with thiobarbituric acid with spectrofluorimetric detection: excitation 515 nm and emission 552 nm. MDA concentration was calculated from the standard curve, prepared from 1,1,3,3-tetraethoxypropane [23].

All dilated cardiomyopathy patients (group D), for purpose of this study, were divided into two groups depending on functional capacity (group A: patient with mild limitation in daily activity, I and II NYHA class, and group B: patients with severe limitation in NYHA III or ambulatory class IV). Additionally redox and inflammatory parameters were analyzed in subgroup of patients according to severity of left ventricle dysfunction (LVEF ≥20%, group A1, and LVEF <20%, group B1).

### 2.4. Statistic

Normality of the distribution of the continuous data was analysed by Shapiro-Wilk test. If the distribution was normal, the data were presented as mean ± standard deviation and were compared with Student's* t*-test. If the distribution was nonnormal the data were presented as median with the first and fourth quartiles and were compared using “*U*” Mann-Whitney test. Categorical data were presented as absolute numbers and percentage and were compared using *χ* ^2^ test. Spearman correlation coefficient was counted for particular parameters. Results were considered statistically significant if *P* < 0.05. Lack of statistical significance was presented as NS (nonsignificant). Statistical analysis was performed with Statistica 10.0 software (Statsoft Inc., Tulsa, USA).

## 3. Results

### 3.1. Clinical and Laboratory Characteristics

One hundred nine patients with heart failure caused by nonischemic DCM aged 45.9 ± 10.8 years old (16 females) were finally enrolled into the study. All patients were clinically stable in the last one month. The twenty-eight healthy control subjects aged 38.1 ± 5.4 (5 females) were not significantly younger than the patients. Of all the patients studied, 26.6% were hypertensive and 11% had type 2 diabetes. The majority of patients were treated with *β*-blockers (94.5%) and either an ACE (92.6%) (angiotensin-converting enzyme) inhibitor and/or an ARB (angiotensin receptor blocker). Most of the patients were treated with diuretics, spironolactone and digoxin (Table 1). The mean time from onset of symptoms of heart failure was 4.59 years and duration of illness did not differ between groups A and B.

Demographic, clinical, and laboratory data in patients with mild and severe limitation of functional capacity were presented in Tables 2 and 3. There were no abnormalities in value of creatinine clearance and hemoglobin concentration. NT-proBNP level was elevated. All the group of DCM patients characterized typical echocardiographic features of impairment of left ventricle systolic function. LVEF was severely depressed, consistent with advanced disease. Hemodynamic measurements showed elevated pulmonary wedge pressure and resulted in mild pulmonary hypertension with elevated PVRI. Simultaneously reduced cardiac index was detected. The reduced SvO_2_ was present (Table 3).

Patients of group B often used loop diuretics and oral anticoagulation (Table 1). There were no differences between groups in demographic and clinical data (Table 2). Lower saturation of mixed venous blood was detected in group B than in group A. NT-proBNP concentration was higher in group B compared to group A (Table 2). Moreover, group B was characterized by worse values of echocardiographic and hemodynamic parameters when compared to group A (Table 3).

### 3.2. Comparison of Redox State and Inflammation in Patients Stratified by NYHA Class

**Figure 1:**
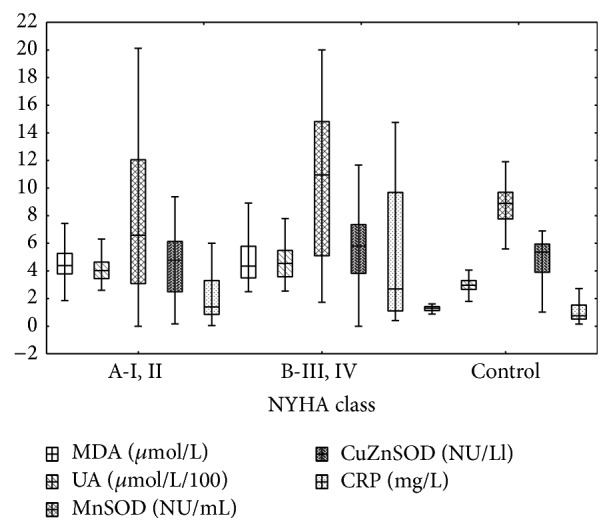
Redox and inflammation biomarkers in I, II and III, IV NYHA class patients and control. Median [25%–75%] and 1.5IQR.

DCM patients presented higher concentrations of MDA and uric acid compared to control (Table 4). There was no difference in MDA concentration between groups A and B. UA concentration was significantly higher in group B than group A (Figure 1).

There were no differences in superoxide dismutase isoenzymes activity between control and all DCM patients (Table 4). But patients of group B presented significant higher MnSOD activity compared to both control and group A patients (Figure 1). hs-CRP concentration was higher in DCM patients compared to the control group and in group B compared to group A (Figure 1).

### 3.3. Comparison of Redox State and Inflammation in Patients Stratified by LVEF

**Figure 2:**
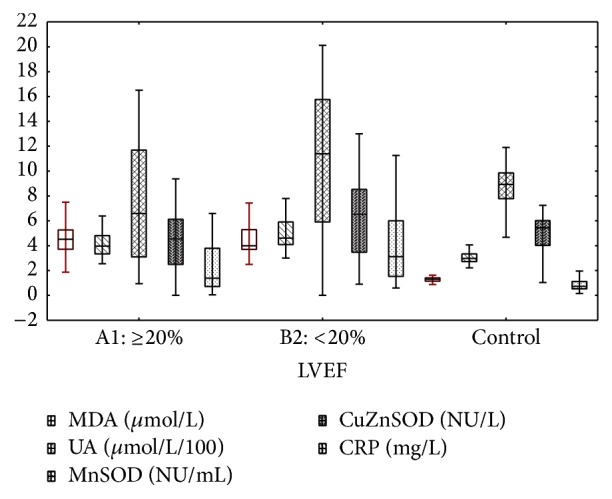
Redox and inflammation biomarkers in patients with LVEF ≥20%, <20%, and control. Median [25%–75%] and 1.5IQR.

Patients group B1 (lower LVEF) had significantly higher concentration of UA, hs-CRP and activity of both izoenzymes SOD in comparison to patients from group A1. MDA concentration was comparable (Figure 2). Except CuZnSOD, patients in B1 group had higher values of examined biomarkers than control (Figure 2, Table 4).

### 3.4. Correlations between Biomarkers and LVEF and Hemodynamic Parameters

#### 3.4.1. Uric Acid

**Figure 3:**
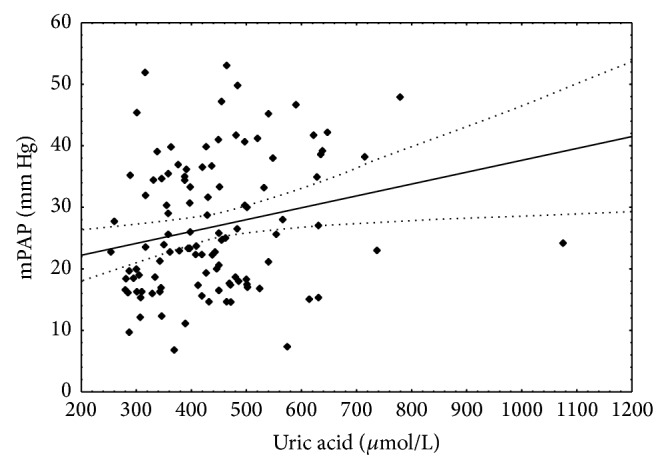
Correlation between uric acid concentration and mean pulmonary artery pressure. Spearman *r* = 0.236; *P* < 0.05.

There were statistically significant positive correlations between uric acid concentration and mPAP (Figure 3) and PVRI. Uric acid concentration negatively correlated with LVEF (Table 5).

#### 3.4.2. hs-CRP

**Figure 4:**
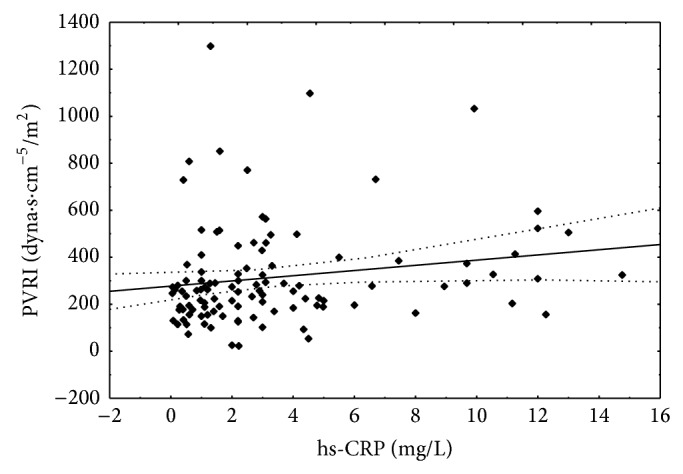
Correlation between hs-CRP concentration and pulmonary vascular resistance index. Spearman *r* = 0.236; *P* < 0.05.

hs-CRP concentration negatively correlated with LVEF. Positive correlations between hs-CRP and PVRI were found (Figure 4, Table 5).

#### 3.4.3. Oxidative Parameters (CuZnSOD, MnSOD, and MDA)

Both isoenzymes' activities positively correlated with mPAP and PWP. The negative correlations between them and LVEF were detected. There were no correlations between MDA concentration with LVEF and examined hemodynamic parameters (Table 5).

### 3.5. Correlations between Biomarkers

**Figure 5:**
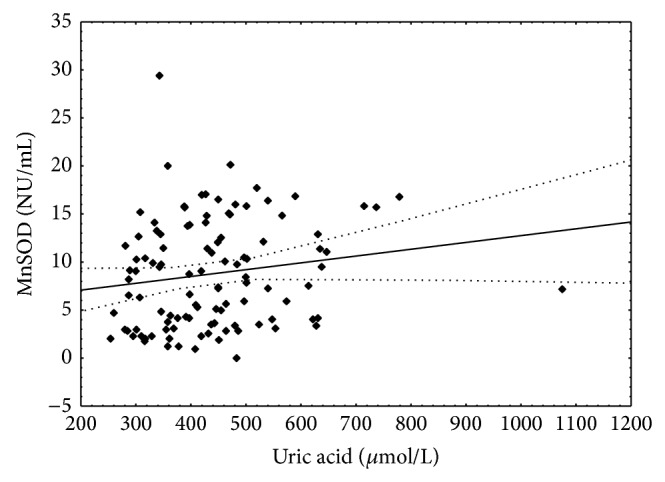
Correlation between uric acid concentration and manganese superoxide dismutase activity. Spearman *r* = 0.22; *P* < 0.05.

SvO_2_ negatively correlated with NT-proBNP concentration which is reflection of heart failure conditions. Additionally negative correlation between SvO_2_ and UA was detected. There is statistically significance positive correlation limit between MnSOD activity and NT-proBNP concentration (*r* = 0.194; *P* = 0.057). MnSOD positively correlated with CuZnSOD (Table 6) and with UA concentration (Figure 5).

**Figure 6:**
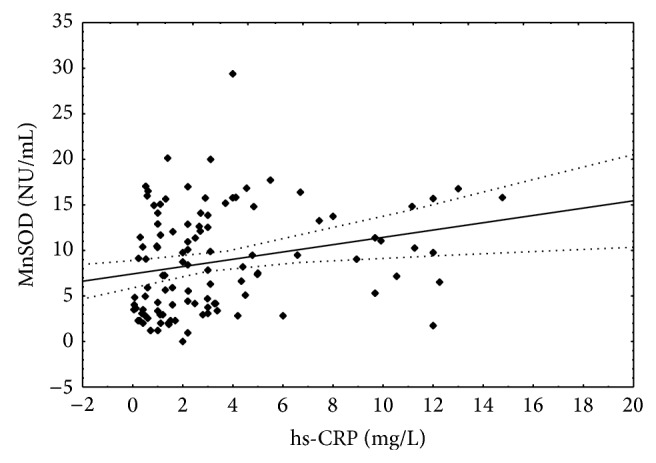
Correlation between hs-CRP concentration and manganese superoxide dismutase activity. Spearman *r* = 0.286; *P* < 0.05.

**Figure 7:**
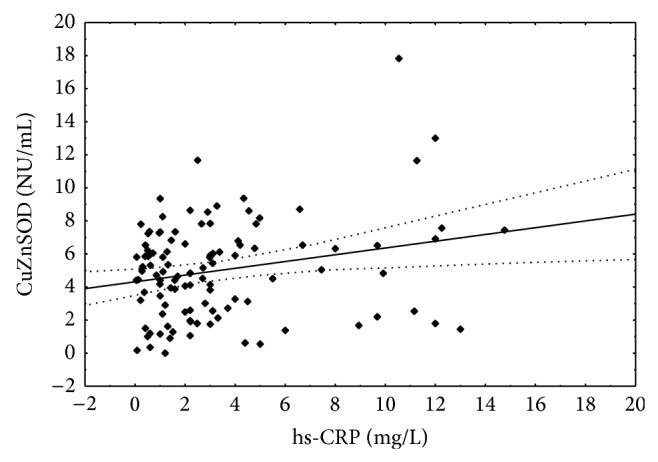
Correlation between hs-CRP concentration and copper-zinc superoxide dismutase activity. Spearman *r* = 0.364; *P* < 0.01.

The positive correlations between CRP concentration and activities of superoxide isoenzymes were found (Figures 6 and 7).

## 4. Discussion

Inflammation and oxidative stress may accompany especially decompensated heart failure and in some cases may be regarded as main cause of dilated cardiomyopathy [24]. In the present study we evaluated 109 patients with severe left ventricle systolic dysfunction, with different grade of symptoms, admitted to the hospital to routine procedure. It should be emphasized that patients were clinically stable and although they did not take antioxidants, they received the optimal treatment of heart failure. Both ACE inhibitors, like some *β*-blockers, have proven antioxidant activity [25, 26]. We appraised the patients with nonischemic etiology of cardiomyopathy to rule out the additional elements in the pathogenesis heart failure like ischemia and inflammation associated with atherosclerosis.

We studied plasma concentration of UA and MDA, activity of SOD isoenzymes, and hs-CRP concentrations. MDA concentration was significantly increased in DCM patient compared to control. In two groups of patients with mild and severe limitation functional capacity (NYHA I, II and NYHA III, IV) we have found no significant differences in MDA level. There was no correlation between MDA level and severity of HF and echocardiographic and hemodynamic parameters. Our results are similar to previous reports which demonstrated increase in MDA level in patients with HF [27, 28]. Opposite to our results in some studies correlations between the plasma level of MDA and indices of HF severity such as NYHA class, LVEF, and ventricular dimension have been shown [29, 30]. Some differences may results from the inclusion in the study patients with HF of different etiology. However, Keith et al. [29] and McMurray et al. [31] found increased MDA level both in patients with HF of ischemic origin and in patients with other causes of HF. In another paper by Tingberg E. there was no change in MDA level and there was a significant correlation between MDA and PWP and no correlation with CI. The mean LVEF in patients included in this study was about twice higher than in our group.

In our DCM group concentration of UA was markedly increased in comparison to the control. The highest value of UA was observed in patient with severe HF. Furthermore, UA level correlated with LVEF and examined hemodynamic parameter without systemic arterial pressure. Recently Borghi et al. also have found inverse relation of serum UA to LVEF in male elderly patients with HF. One of the potential mechanisms which may explain this result is lower cardiac index leading to hypoxemia. There was a slight correlation between UA and SvO_2_ as indicator of tissue hypoxia in our study [32]. Leyva et al. have not observed correlation with LEVF but similar to us they have indicated the relationships between UA concentration and the functional capacity (NYHA class and maximal oxygen uptake) in patients with cardiac failure [33]. Previous studies have shown increased UA level not only in HF patients but also in patients with tissue hypoxemia due to obstructive sleep apnea or COPD [34, 35]. Accepted source of elevated UA in HF patients is breakdown of ATP to adenosine and hipoxanthine and increase in the generation of uric acid by xanthine dehydrogenase and xanthine oxidase. In addition, lactic acid generated during hypoxia can result in the urinary excretion of lactate that increases the absorption of urate in the proximal tubule [36]. UA concentration is known as a predictor of poor prognosis in heart failure patient [37]. During increased UA production we have observed increased ROS production and mitochondrial Mn-SOD activity. Increased ROS production connected with increased UA concentration results in increased Mn-SOD activity.

Previous studies by Hill and Singal demonstrated that HF subsequent to myocardial infarct was associated with the antioxidant decrease as well as increased oxidative stress [38]. In contrast there was no decrease in SOD activity in study by Tsutsui et al. [39]. Some results indicated that oxidative stress in HF might be primarily due to the enhancement of ROS generation rather than to the decline in antioxidant defense within the heart [39].

Our study demonstrates the elevated plasma SOD activity, mainly MnSOD in NYHA III-IV patients comparing to the I-II NYHA patients and control group. There were no changes in Cu,Zn-SOD activity between studied groups divided depending on NYHA class and control. Both MnSOD and CuZnSOD activity were higher in patients with lower LVEF.

For prevention the oxidative stress in heart failure patients we have seen adaptational increase of the enzymatic antioxidative defense represent by increase in MnSOD and CuZnSOD activity in group with LVEF <20% comparing to the group LVEF ≥20%. Comparing with the control group we have seen decrease of SOD activity in NYHA I, II and LVEF ≥20% group. This transition from decrease to increase in SOD activity in our HF patients may be the key factor providing a constant MDA level. Some different adaptational changes with increase of the antioxidative defense followed by the formation of a relative deficit were found by Schimke et al. in myocardial tissue of heart transplant recipients [20]. The importance of enhanced antioxidant activity to protect the contractile function of the surviving myocardium against the damaging influence of hypoxia/reoxygenation during the postinfarct period was indicated by Wagner et al. in rat's myocardial infarction model [40, 41].

Our data do not show the tissue origins of the markers of increased lipid peroxidation or SOD; both poorly perfused peripheral muscles and the myocardium could have contributed [42]. Lack of correlation between MDA and severity of HF is surprising.

The function of antioxidant system is not to remove these oxidants entirely but instead to keep them at the level below which they will trigger the inflammatory cascade, a series of intracellular and intranuclear signaling that results in the release of destructive inflammatory cytokines [43]. It has became evident that heart failure is associated with subclinical inflammation which is in agreement with study demonstrating non-specific elevation in levels of some proinflamatory markers, such CRP, TNF-alfa, Il-6 [24].

The positive correlation between CRP concentration and activities of superoxide isoenzymes suggests the existence of link between free radicals and inflammation in DCM. Rankinen observed additionally correlation between MDA and hs-CRP but we did not observe such correlation [44].

In our study we have observed increase in CRP level according to the NYHA class and LVEF. It was the same as the results obtained by Alonso-Martínez et al. [45]. An association between oxidative stress state parameters and inflammation markers could be explained through the action of cytokines that play a pivotal role in hs-CRP biosynthesis.

Unfortunately, we only have a number of pieces of the puzzle, but these pieces cannot yet be connected to provide final pathway of relationships between oxidative stress and inflammation. It would be valuable information to determine if examined parameters are capable of predicting prognosis in patients with chronic heart failure (CHF).

## 5. Conclusion

The correlation between MnSOD and mPAP, PWP, LVEF combined with correlation between MnSOD and NT-proBNP, CRP, and UA may indicate a link among increased mitochondrial ROS generation, severity of HF, systemic and pulmonary hemodynamic, and the level of inflammation in DCM patients. Thus it is plausible to think that mitochondria are an important source of ROS in the HF.

## Figures and Tables

**Table 1:** Pharmacological treatment with division into groups depending on NYHA class.

	All DCM (D) *n* = 109	D (%)	NYHA I-II (A) *n* = 66	A (%)	NYHA III-IV (B) *n* = 43	B (%)	*χ* ^2^
BB	103	94.50	61	92.42	42	97.67	NS
ACE-I	101	92.66	62	93.94	39	90.70	NS
ARB	39	35.78	26	39.39	13	30.23	NS
MRA	99	90.83	59	89.39	40	93.02	NS
AM	15	13.76	9	13.64	6	13.95	NS
LD	66	60.55	31	46.97	35	81.40	*P* < 0.001
TD	20	18.35	14	21.21	6	13.95	NS
OAK	49	44.95	24	36.36	25	58.14	*P* < 0.05
DIG	69	63.30	39	59.09	30	69.77	NS

BB: *β*-blocker; ACE-I: angiotensin converting enzyme inhibitors; ARB: angiotensin receptor blockers; MRA: mineralocorticoid receptor antagonist; AM: amiodarone; LD: loop diuretic; TD: thiazide diuretic; OAK: oral anticoagulation; DIG: digitalis.

**Table 2:** Clinical and selected laboratory results in all patients and with division into groups depending on NYHA class.

	All DCM (D) *n* = 109	NYHA I-II (A) *n* = 66	NYHA III-IV (B) *n* = 43	A versus B
NYHA functional class I/II/III/IV *n* (%)	3/63/34/9 (2.7/57.8/31.2/8.3)	3/63 (4.5/95.5)	34/9 (79/21)	
Sex M/F *n* (%)	91/16 (83.5/16.5)	53/13	40/3	NS
Age Y $\overset{-}{X}$ (±SD)	45.9 ± 10.8	47.2 ± 10.2	43.9 ± 11.6	NS
BMI $\overset{-}{X}$ (±SD)	28.9 ± 18.4	30.1 ± 14.8	27.0 ± 4.3	NS
Duration of illness	4.6 ± 4.2	4.9 ± 4.5	4.1 ± 3.7	NS
Hypertension *n* (%)	29 (26.6)	18	11	NS
Diabetes mellitus *n* (%)	12 (11.0)	6	6	NS
NT-proBNP [pg/mL]	820.5 346.5–1837	582.5 209.5–1883	2045 1045–3000	*P* < 0.001
Hemoglobin [g/L]	134.2 ± 32.0	138.3 ± 26.5	127.6 ± 40.8	NS
Creatinine clearance [mL/min]	123.0 ± 53.8	130.1 ± 47.3	111.4 ± 32.8	NS

NYHA: New York Heart Association functional class; BMI: body mass index; NT-proBNP: N-terminal pro-B-type natriuretic peptide.

**Table 3:** Results of selected echocardiographic and haemodynamic parameters in all patients and with division into groups depending on NYHA class.

	All DCM (D) *n* = 109	NYHA I-II (A) *n* = 66	NYHA III-IV (B) *n* = 43	A versus B
LVEF (%)	22.94 ± 7.10	25.13 ± 7.17	19.45 ± 6.99	*P* < 0.001
LVEDD [mm]	68.71 ± 10.85	67.27 ± 12.04	71.00 ± 8.96	*P* < 0.05
mPAP [mm Hg]	26.99 ± 9.66	23.14 ± 8.98	33.12 ± 10.74	*P* < 0.001
mABP [mm Hg]	92.21 ± 13.65	95.15 ± 14.40	87.53 ± 12.60	*P* < 0.01
PWP [mm Hg]	19.05 ± 8.34	15.79 ± 7.82	24.26 ± 9.17	*P* < 0.001
PVRI [dyna*·*s*·*cm^−5^/m^2^]	314.79 ± 212.1	270.4 ± 190.0	385.6 ± 247.4	*P* < 0.01
CI [L/min/m^2^]	2.19 ± 0.53	2.32 ± 0.44	2.01 ± 0.66	*P* < 0.01
SvO_2_ [%]	57.57 ± 10.99	59.32 ± 9.83	54.76 ± 12.84	*P* < 0.05

LVEF: left ventricle ejection fraction; LVEDD: left ventricle end-diastolic diameter; mPAP: mean pulmonary artery pressure; mABP: mean arterial blood pressure; PWP: pulmonary wedge pressure; PVRI: pulmonary vascular resistance index; CI: cardiac index; SvO_2_: mixed venous blood saturation.

**Table 4:** Redox biomarkers and hs-CRP in healthy controls and all DCM patients and with division of them into groups depending on NYHA class and LVEF.

	Control C (*n* = 28)	DCM D (*n* = 109)	NYHA I-II A (*n* = 66)	NYHA III-IV B (*n* = 43)	*P* “*U*” Mann-Whitney T	LVEF ≥20% A1 (*n* = 74)	LVEF <20% B1 (*n* = 35)	*P* “*U*” Mann-Whitney T
MDA [*μ*mol/L]	1.31 1.14–1.41	4.37 3.68–5.78	4.47 3.71–5.61	4.26 3.51–5.78	^ AvsB^ns ^AvsC^<0.001 ^BvsC^<0.001 ^DvsC^<0.001	4.51 3.71–5.27	4.00 3.70–5.29	^ A1vsB1^ns ^A1vsC^<0.001 ^B1vsC^<0.001

UA [*μ*mol/L]	296 266–330	427 345–500	397 331–471	462,0 358–566	^ AvsB^<0.05 ^AvsC^<0.001 ^BvsC^<0.001 ^DvsC^<0.001	398 334–480	462 409–590	^ A1vsB1^<0.01 ^A1vsC^<0.001 ^B1vsC^<0.001

MnSOD [NU/mL]	8.89 7.76–9.70	9.04 4.16–13.74	7.23 3.49–12.65	10.52 5.91–14.82	^ AvsB^<0.05 ^AvsC^ns ^BvsC^<0.05 ^DvsC^ns	6.58 3.09–11.69	11.39 5.92–15.76	^ A1vsB1^<0.001 ^A1vsC^ns ^B1vsC^<0.05

CuZnSOD [NU/mL]	5.37 3.09–5.95	4.88 2.60–6.79	4.72 2.49–6.1	5.92 3.88–7.3	^ AvsB^ns ^AvsC^ns ^BvsC^ns ^DvsC^ns	4.55 2.49–6.12	6.52 3.47–8.53	^ A1vsB1^<0.05 ^A1vsC^ns ^B1vsC^ns

hs-CRP [mg/L]	0.76 0.53–1.53	1.7 0.71–4.34	1.45 0.47–3.27	2.7 1.11–9.68	^ AvsB^<0.05 ^AvsC^<0.05 ^BvsC^<0.01 ^DvsC^<0.05	1.85 0.71–3.10	3.29 2.00–5.70	^ A1vsB1^<0.01 ^A1vsC^<0.01 ^B1vsC^<0.001

MDA: malondialdehyde; UA: uric acid; MnSOD: manganese superoxide dismutase; CuZnSOD: cooper-zinc superoxide dismutase; hs-CRP: high sensitivity C-reactive protein.

**Table 5:** Spearman *r* correlation between biomarkers and left ventricle ejection fraction and hemodynamic parameters.

	NT-proBNP	Uric acid	CRP	SvO_2_	MnSOD	CuZnSOD	MDA
LVEF	−0.547 *P* < 0.001	−0.285 *P* < 0.01	−0.240 *P* < 0.05	0.027 NS	−0.251 *P* < 0.05	−0.190 *P* = 0.051	0.114 NS

mPAP	0.548 *P* < 0.001	0.236 *P* < 0.05	0.161 NS	−0.262 *P* < 0.05	0.252 *P* < 0.01	0.243 *P* < 0.05	−0.029 NS

mABP	−0.373 *P* < 0.001	0.114 NS	−0.225 NS	0.191 NS	−0.085 NS	0.021 NS	−0.039 NS

PWP	0.563 *P* < 0.001	0.167 NS	0.084 NS	−0.224 *P* < 0.05	0.239 *P* < 0.05	0.247 *P* < 0.05	−0.037 NS

PVRI	0.252 *P* < 0.05	0.236 *P* < 0.05	0.236 *P* < 0.05	−0.354 *P* < 0.001	0.096 NS	0.015 NS	0.014 NS

SVRI	0.177 NS	0.194 NS	−0.218 NS	−0.262 *P* < 0.05	−0.012 NS	0.035 NS	−0.098 NS

CI	−0.544 *P* < 0.001	−0.193 NS	0.072 NS	0.428 *P* < 0.001	−0.064 NS	−0.084 NS	0.079 NS

NT-proBNP: N-terminal pro-B-type natriuretic peptide; CRP: C-reactive protein; SvO_2_: mixed venous blood saturation; MnSOD: manganese superoxide dismutase; CuZnSOD: cooper-zinc superoxide dismutase; MDA: malondialdehyde; LVEF: left ventricle ejection fraction; mPAP: mean pulmonary artery pressure; mABP: mean arterial blood pressure; PWP: pulmonary wedge pressure; PVRI: pulmonary vascular resistance index; SVRI: systemic vascular resistance index; CI: cardiac index.

**Table 6:** Spearman *r* correlation between biomarkers.

	NT-ProBNP	Uric acid	CRP	SvO_2_	MnSOD	CuZnSOD	MDA
NTproBNP		0.167 NS	0.062 NS	−0.388 *P* < 0.001	0.194 *P* = 0.057	0.154 NS	−0.058 NS

SvO_2_	−0.388 *P* < 0.001	−0.192 NS	0.189 NS		−0.142 NS	0.102 NS	−0.062 NS

Uric acid	0.167 NS		0.188 NS	−0.192 NS	0.220 *P* < 0.05	0.017 NS	−0.021 NS

CRP	0.062 NS	−0.010 NS		0.189 NS	0.286 *P* < 0.05	0.364 *P* < 0.01	0.117 NS

MnSOD	0.194 *P* = 0.057	0.220 *P* < 0.05	0.286 *P* < 0.05	−0.142 NS		0.344 *P* < 0.001	0.080 NS

CuZnSOD	0.154 NS	0.017 NS	0.364 *P* < 0.01	0.102 NS	0.344 *P* < 0.001		−0.057 NS

MDA	−0.058 NS	−0.021 NS	0.117 NS	−0.062 NS	0.080 NS	−0.057 NS	

NT-proBNP: N-terminal pro-B-type natriuretic peptide; CRP: C-reactive protein; SvO_2_: mixed venous blood saturation; MnSOD: manganese superoxide dismutase; CuZnSOD: cooper-zinc superoxide dismutase; MDA: malondialdehyde.
